# SARS-CoV-2 one year on: evidence for ongoing viral adaptation

**DOI:** 10.1099/jgv.0.001584

**Published:** 2021-04-15

**Authors:** Thomas P. Peacock, Rebekah Penrice-Randal, Julian A. Hiscox, Wendy S. Barclay

**Affiliations:** ^1^​ Department of Infectious Diseases, St Marys Medical School, Imperial College London, UK; ^2^​ Institute of Infection, Veterinary and Ecology Sciences, University of Liverpool, UK; ^3^​ A*STAR Infectious Diseases Laboratories (A*STAR ID Labs), Agency for Science, Technology and Research (A*STAR), Singapore

**Keywords:** SARS-CoV-2, COVID-19, coronavirus, mutant, adaptation, pandemic

## Abstract

SARS-CoV-2 is thought to have originated in the human population from a zoonotic spillover event. Infection in humans results in a variety of outcomes ranging from asymptomatic cases to the disease COVID-19, which can have significant morbidity and mortality, with over two million confirmed deaths worldwide as of January 2021. Over a year into the pandemic, sequencing analysis has shown that variants of SARS-CoV-2 are being selected as the virus continues to circulate widely within the human population. The predominant drivers of genetic variation within SARS-CoV-2 are single nucleotide polymorphisms (SNPs) caused by polymerase error, potential host factor driven RNA modification, and insertion/deletions (indels) resulting from the discontinuous nature of viral RNA synthesis. While many mutations represent neutral ‘genetic drift’ or have quickly died out, a subset may be affecting viral traits such as transmissibility, pathogenicity, host range, and antigenicity of the virus. In this review, we summarise the current extent of genetic change in SARS-CoV-2, particularly recently emerging variants of concern, and consider the phenotypic consequences of this viral evolution that may impact the future trajectory of the pandemic.

## Introduction

Towards the end of 2019, reports began of an unknown respiratory illness in the Chinese city of Wuhan. Within several weeks, it became clear these infections were being caused by a SARS-like coronavirus, which was termed SARS-CoV-2, with the associated disease called COVID-19. In severe cases this results in extensive immunopathology in the lungs [1]. By early March 2020, the virus had entered many countries across the world and the WHO declared a pandemic on 11 March [2]. In the months since, different countries across the world have enacted different pandemic response plans that vary from recurrent lockdowns, mask mandates, social distancing rules, or uncontrolled circulation in a hope to acquire herd immunity. In areas with elevated SARS-CoV-2 prevalence, high levels of morbidity and excess mortality, particularly in the elderly, has resulted. As of 13 March 2021, there have been an estimated 120 million confirmed cases of COVID-19 globally with over 2.6 million confirmed deaths [3].

**Fig. 1.**
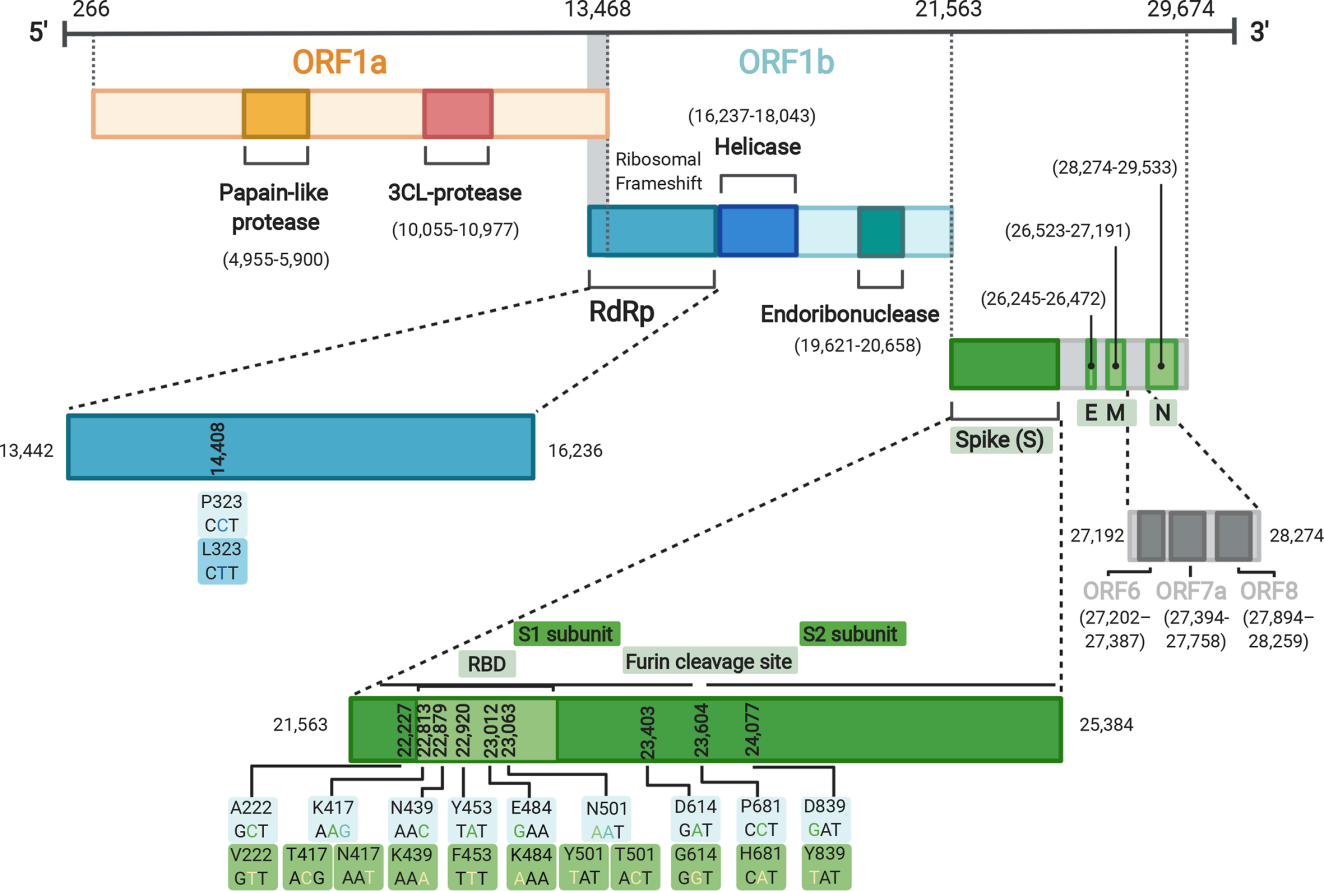
Genome organisation of SARS-CoV-2 with regions of interest annotated. Mutations of interest (for example those found in B.1.1.7) shown as both nucleotide and amino acid changes. Figure made using Biorender (https://biorender.com/).

SARS-CoV-2 is a betacoronavirus, containing a ~30 kb positive-sense RNA genome, among the largest of any RNA virus (Fig. 1). Coronaviruses, such as SARS-CoV-2, avoid error catastrophe by encoding an exoribonuclease (nsp14) that confers a unique proofreading mechanism during viral RNA synthesis [4, 5]. Genome sequencing of SARS-CoV-2 throughout the course of the outbreak, has revealed a nucleotide substitution rate of ~1×10^−3^ substitutions per year [6]. This is comparable to the substitution rate observed for Ebola virus (1.42×10^−3^) during the 2013–2016 West African outbreak [7]. However, SNPS are not the only genetic variation seen commonly in coronaviruses.

Replication of the coronavirus genome and transcription of viral subgenomic mRNAs (sgmRNAs) are complex processes. The genome is roughly organised into two regions. The first two thirds of the genome is immediately translated and proteolytically processed in the host cell cytoplasm to generate the viral polymerase/transcriptase complex and other viral proteins. The remaining one third of the genome is expressed and translated through a nested set of sgmRNAs, this includes the spike glycoprotein and other structural and accessory proteins. These sgmRNAs are 5′ and 3′ co-terminal with the genome; the 5′ end contains a leader sequence that is present on the 5′ end of the genome. Along the genome, proceeding each ORF is a transcription regulatory sequence (TRS). The prevailing thought is that an integral part of the transcription mechanism in coronaviruses for the synthesis of viral sgmRNAs involves a discontinuous step. The easiest way to visualise this, is that the polymerase/transcriptase complex binds to the 3′ end of the positive strand and proceeds along the genome in a 3′ to 5′ direction synthesizing a negative strand. When the polymerase/transcriptase complex reaches a TRS, the newly synthesized negative strand can translocate to the 5′ leader sequence of the genome where it is then copied. This forms a negative sense sgmRNA that is then copied into the positive sense sgmRNA [8]. This discontinuous nature has the consequence of a high degree of recombination resulting in the insertion of viral and non-viral sequences into - or frequent deletions of viral sequence from - the genome. This can result in the formation of viable genomes as well as defective interfering RNAs. Therefore, both SNPs and indels are likely to be the major processes allowing coronaviruses to rapidly switch host range or change their pathogenicity and/or virulence. For example, in cats infected by feline enteric coronavirus (FECV), variants can be generated within an infected animal by deletion of a key furin cleavage site in the spike protein. This results in feline peritonitis virus (FIPV) that causes a systemic fatal disease [9].

Recombination between different coronaviruses has been hypothesised to have given rise to both the genetically divergent receptor binding domain of SARS-CoV-2 spike [10, 11], as well as the insertion of the S1/S2 furin (polybasic) cleavage site [12]. MERS-CoV is also thought to have had a major recombination event in recent evolutionary history [13]. Furthermore, deletions in the genome of the porcine coronavirus transmissible gastroenteritis virus (TGEV) gave rise to a new virus called porcine respiratory coronavirus (PRCV) [14]. Human seasonal coronavirus HCoV-OC43 and -HKU1 are thought to have acquired a hemagglutinin esterase (HE) gene following recombination between a progenitor coronavirus and influenza C-like virus [15]. Variants of OC43 and HKU1 HE have been shown to lose their sialic acid binding activity through progressive deletions in their lectin domains [16]. Finally, the N-terminal domain (NTD) of coronavirus spike proteins shares a number of structural similarities to eukaryotic galectins, leading to some to hypothesise the precursor to coronaviruses may have incorporated a portion of the host gene in the distant past [17]. Host RNA may also be a source for the polybasic cleavage site, similar to the proposed mechanism for generating highly pathogenic avian influenza viruses [18]. Studies on a recombinant attenuated SARS-CoV lacking the envelope (E) gene or the PDZ-binding motif (generated as a potential vaccine candidate) showed the virus could revert to virulence by partially duplicating a viral sequence (from ORF8a) which restored E function [19].

### The spike protein is the major entry protein and antigen of SARS-CoV-2

**Fig. 2.**
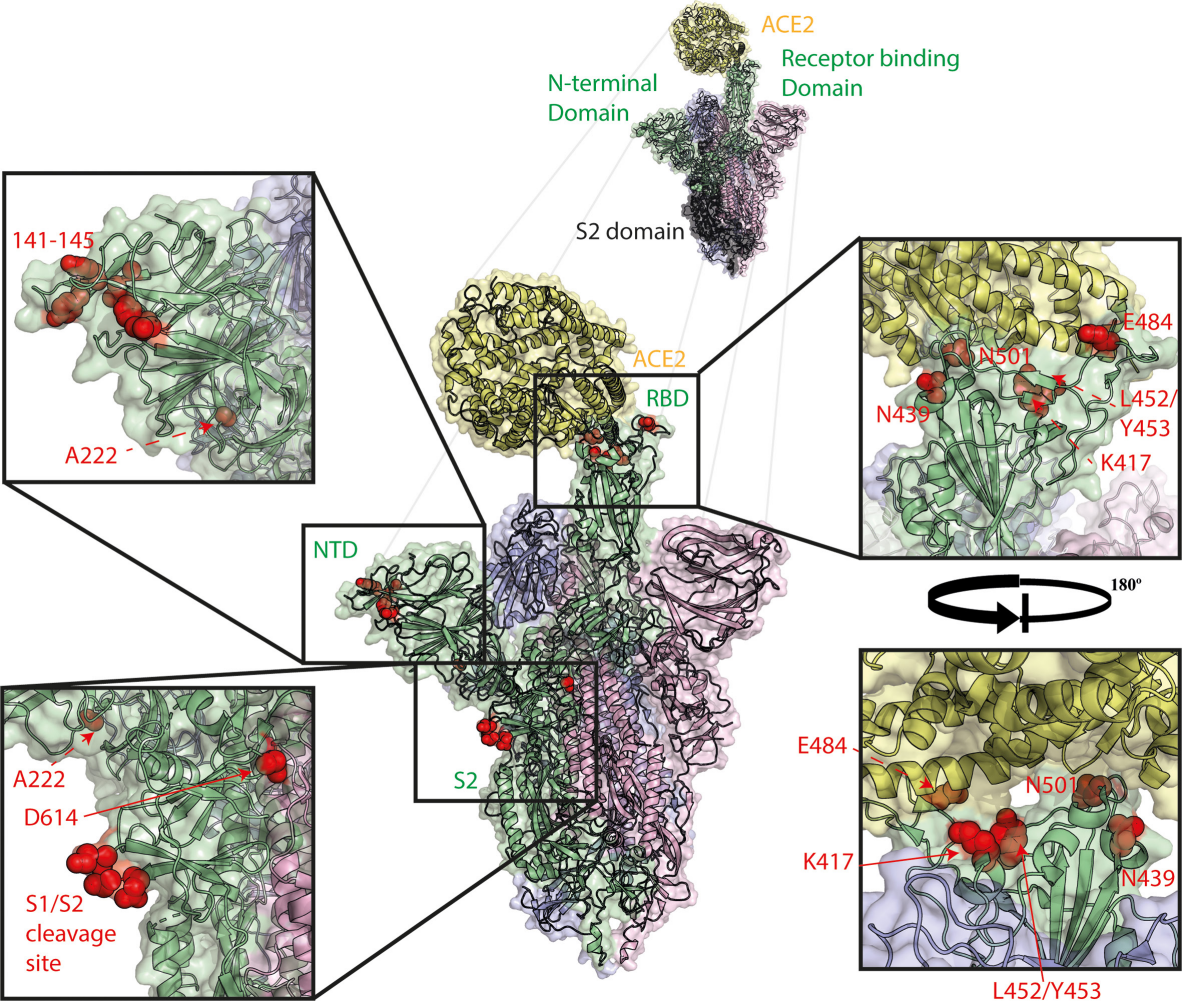
Spike mutations of interest mapped to the spike trimer. Mutations shown in red, ACE2 shown in yellow, spike monomer in RBD ‘up’ conformation shown in green, spike monomers in RBD ‘down’ conformation shown in pink and blue. Structure made using PyMOL using PDBID 7A94 [24].

Spike is the major glycoprotein responsible for SARS-CoV-2 entry, as well as the primary antigen and target of most SARS-CoV-2 vaccines currently in use and future development (Fig. 2). SARS-CoV-2 virions contain approximately 23 spike trimers on their surfaces [20]. The SARS-CoV-2 spike glycoprotein is synthesised as a single precursor polypeptide that forms trimers. Spike is subsequently cleaved into two major subunits, S1 and S2, by endogenous cellular furin [21]. The S1 subunit is composed of two further subdomains – an N-terminal domain (NTD), whose function is poorly described for SARS-CoV-2 but can act as a receptor binding domain in some coronaviruses and a potential glycan shield against antibody-mediated immunity, and a C-terminal receptor binding domain (RBD). The RBD of SARS-CoV-2 (as with SARS-CoV and seasonal HCoV-NL63) binds human angiotensin-converting enzyme 2 (ACE2), as its cognate cell surface receptor [22]. Spike glycoprotein shifts between two separate conformations – an ‘open’ or ‘up’ conformation able to effectively bind ACE2, and a ‘closed’ or ‘down’ conformation, with its receptor binding interface packed down into the top of the spike trimer [23, 24]. Different trimers may have one, two or three spike glycoproteins in either conformation. It has been suggested that the closed conformation may allow for viral escape from RBD-binding neutralising antibodies. The S2 subunit contains the spike fusion peptide, a transmembrane domain and a short cytoplasmic tail. This short cytoplasmic tail contains a signal sequence that retains the spike in the endoplasmic reticulum from where, after particle assembly, virions are able to bud into the endoplasmic reticulum-Golgi intermediate compartment (ERGIC) [25]. Immediately adjacent to the fusion peptide is a second protease cleavage site termed the S2’ cleavage site. Upon both S1/S2 cleavage and receptor binding by the RBD, the S1 subunit dissociates from S2 exposing the S2’ site and enabling its cleavage by cellular proteases such as TMPRSS2 or Cathepsin L [26–28]. S2’ cleavage results in immediate activation of the fusion peptide and subsequent spike-mediated membrane fusion [26].

Due to the spike glycoprotein being the major viral antigen and since the RBD/ACE2 interaction is a major host range determinant, there is considerable selection pressure placed on this region of the viral genome. Generally, the S1 subunit is thought to be the major inducer of a protective antibody response and variation in this region can result in antigenic drift either against previous infection by other variants or induced by vaccination [29].

### D614G is now found in the majority of SARS-CoV-2 isolates and enhances virus infectivity and transmissibility in humans

The best characterised of the polymorphisms seen in SARS-CoV-2 since its emergence is the spike glycoprotein mutation D614G. Viruses with D614G were first detected in February 2020 and by May, around 80 % of sequences globally were found to contain this mutation [30–32]. The rapid replacement of previously circulating SARS-CoV-2 strains is likely due to this virus being slightly more transmissible than the previous strains combined with a strong founder effect as the virus exponentially expanded in a first pandemic wave across Europe and the Americas [30, 33]. Notably, the major clade containing D614G (Pango lineage B.1 and its sub-lineages) also contained several other genetically linked mutations, including one in the main polymerase subunit NSP12, P323L, that may also have contributed to its dominance by exerting a fitness advantage. On the other hand, there are several examples of independent acquisition of D614G (but not P323L), such as the A.19 and A.2.4 lineages, which continue to circulate [34]. D614G has now been shown by a multitude of independent studies to enhance entry into human ACE2 expressing cells in pseudovirus based assays *in vitro* [30, 32, 35–37]. In addition, several studies linked D614G containing viruses to lower Ct values in clinical SARS-CoV-2 diagnostic PCR tests, indicating the virus replicated more efficiently in the human respiratory tract, although without any link to higher pathogenicity or severe clinical outcomes [30, 34, 38–40]. Several groups have also shown that D614G containing viruses (either recombinant or naturally occurring strains) had enhanced growth in primary human airway cells and replicated with greater efficiency in animal models such as hamster, ferret or the human ACE2-expressing mouse, and transmitted more efficiently in a hamster model, suggesting D614G alone is sufficient to confer this advantage in the absence of P323L [41–45].

Several non-mutually exclusive mechanisms have been proposed to explain how D614G enhances entry, replication, and transmission. The best described of which is that the polymorphism weakens an interaction at the trimer interface leading to a greater proportion of spike RBD in the ‘open’ conformation and subsequently allows enhanced ACE2 binding [24, 46–50]. An alternative proposed mechanism, though not necessarily incompatible with that previously described, is that D614G stabilises the pre-fusion structure of the spike trimer, preventing premature shedding of the S1 subdomain which can occur after furin cleavage [32, 47, 49, 51]. One additional proposed mechanism, again not necessarily incompatible with the others, is that D614G results in changes in the conformation of the S1/S2 cleavage site loop allowing more efficient access by furin and therefore more efficient S1/S2 cleavage [42, 49, 51, 52].

Once it became clear D614G containing variants were rapidly expanding globally, a major concern was that this might affect the efficacy of vaccines that were being developed which universally contained spike antigens with the ancestral D614. This concern has been allayed by the repeated finding that the D614G variants are equally, if not more readily, neutralised by antisera raised against D614 containing virus or vaccines, as well as by therapeutic monoclonal antibodies [30, 32, 37, 41, 44, 46, 48].

At present, global sequencing surveillance suggests that viruses without D614G are almost non-existent. One exception is a lineage of viruses identified in Uganda (A.23) that, as of March 2021, continued to contain D614. This lineage, however, does contain a nearby spike mutation, Q613H. Although Q613H is currently uncharacterised, it may play a similar role to the D614G substitution, allowing this variant to continue co-circulating [53, 54].

### Y453F is a mink adaptation that allows partial escape from antisera

Ferrets, which are members of the Mustelidae family, have traditionally been models for influenza virus transmission and infection and were quickly utilised in a similar manner for SARS-CoV-2 research, showing a dose dependent response to SARS-CoV-2 and protection from reinfection [55, 56]. Mink are closely related to ferrets and are farmed in many countries for their fur. It became apparent by the middle of 2020 that mink, like ferrets, were highly susceptible to reverse-zoonotic SARS-CoV-2 infection [57, 58]. Although mink (and ferrets) could be readily infected, several spike glycoprotein mutations rapidly and repeatedly arose in these hosts, both in the field and under laboratory conditions, most commonly Y453F and N501T in the RBD (Figs 1 and 2) [59–61]. Both Y453F and N501T have been shown to allow stronger binding of the spike RBD to human ACE2. Moreover, from analysis of the interaction between the spike glycoprotein and ACE2, it is apparent that the Y453F mutation may optimise an interaction with Y34 present in mink and ferret ACE2 [62–64]. Further alarm was raised when a large cluster of human cases were detected in Denmark and the Netherlands which harboured these mutations. In particular, the Y453F mutation was detected in Northern Denmark in combination with several other spike mutations including the NTD deletion Δ69–70 [60, 61]. This virus variant, known as ‘Cluster 5’, was shown to partially escape neutralisation by convalescent antisera [61, 65, 66]. This led to the culling of nearly 17 million mink [67], with several other countries making plans to close their own mink farms or carry out mass culling as a precaution.

It is possible that by adapting its suboptimal interaction with ACE2 protein found in mustelids, the virus may have inadvertently selected for stronger receptor binding to human ACE2 and this may account for loss of neutralisation. It is well described for both human and avian influenza viruses that increases in receptor binding can allow non-specific antibody escape. A stronger interaction between the virus glycoprotein and host receptor may better outcompete weaker competitive binding between antibody and virus glycoprotein [68–70].

Additionally, Y453F has been reported in a single case from an immunocompromised patient, potentially as an adaptation to human ACE2 (see Table 1) [62, 71].

**Table 1.** Summary of substitutions seen in SARS-CoV-2 isolates from immunocompromised patients

Protein	Mutation	References
	N440K	Truong *et al.*
	Y453F	Bazykin *et al.*
	T470N	Bazykin *et al.*
	S477N	Khatamzas *et al.*
	T478K	Choi *et al.*
Spike- RBD	V483A*	Avanzato *et al*., Truong *et al.*
	**E484K/A/Q**	Choi *et al*., Khatamzas *et al*., Truong *et al.*
	F486I	Choi *et al.*
	Y489H	Choi *et al.*
	Q493K	Choi *et al.*
	S494P	Choi *et al.*
	N501Y	Choi *et al.*
	V3G	Borges *et al.*
	P9L	Choi *et al.*
	Δ12–18	Choi *et al.*
	S13I	Truong *et al.*
	Δ18–30	Borges *et al.*
	T22I	Truong *et al.*
	**S50L**	Bazykin *et al*., Borges *et al.*
	W64G	Kemp *et al.*
	**Δ69–70**	Bazykin *et al*., Kemp *et al.*
	V70F	Truong *et al.*
Spike-NTD	N87S	Borges *et al.*
	T95I	Truong *et al.*
	K97M	Truong *et al.*
	**Deletions in 140 region**	Bazykin *et al*., McCarthy *et al*., Avanzato *et al*., Choi *et al*., Khatamzas *et al*., Borges *et al*., Truong *et al*. (3/3 patients)
	Q183H	Choi *et al.*
	R190K	Truong *et al.*
	I197T	Truong *et al.*
	Y200H	Kemp *et al.*
	Δ211/L212I	Truong *et al.*
	N211K	Truong *et al.*
	A222V	Borges *et al.*
	T240I	Kemp *et al.*
	P330S	Kemp *et al.*
	R685Q (furin site)	Avanzato *et al.*
	D737G	Bazykin *et al.*
Spike-other	D796H	Kemp *et al.*
	I870V	Choi *et al.*
	I1020S	Choi *et al.*
	N1108T	Truong *et al.*
	T78I	Truong *et al.*
	G82S	Truong *et al.*
NSP1	Δ85	Avanzato *et al.*
	V86I	Truong *et al.*
	G98V	Khatamzas *et al.*
	R124C	Truong *et al.*
	I21V	Choi *et al.*
	K489E	Khatamzas *et al.*
NSP2	I513T	Kemp *et al.*
	C540F	Truong *et al.*
	E617A	Truong *et al.*
	P74A	Truong *et al.*
	G282V	Khatamzas *et al.*
	**T504A/I**	Bazykin *et al*., Khatamzas *et al.*
	I508V	Khatamzas *et al.*
NSP3	H682Y	Truong *et al.*
	D821N	Bazykin *et al.*
	Δ1267–9	Borges *et al.*
	S1375F	Borges *et al.*
	L1870F	Truong *et al.*
	T204I	Truong *et al.*
	**T295I**	Bazykin *et al*., Borges *et al*. - lineage defining???
	A307V	Choi *et al.*
NSP4	V315I	Bazykin *et al.*
	S386F	Truong *et al.*
	N396S	Khatamzas *et al.*
	T492I	Truong *et al.*
NSP5	T21I	Khatamzas *et al.*
	L89F	Truong *et al.*
	L37F	Truong *et al.*
NSP6	M86I	Truong *et al.*
	M100V	Truong *et al.*
	Q160R	Khatamzas *et al.*
NSP7	V58G	Bazykin *et al.*
NSP9	T24I	Khatamzas *et al.*
	V157L	Kemp *et al.*
NSP12	R457C	Truong *et al.*
	E796D	Truong *et al.*
	P77L	Truong *et al.*
	T115I	Choi *et al.*
	T214I	Truong *et al.*
NSP13	P238L	Truong *et al.*
	V349L	Truong *et al.*
	P504L	Avanzato *et al.*
	Y541C	Avanzato *et al.*
NSP14	E453D	Truong *et al.*
	S461P	Avanzato *et al.*
NSP15	N177S	Kemp *et al.*
	A255V	Truong *et al.*
NSP16	A34V	Borges *et al.*
	S166A	Choi *et al.*
ORF3a	Q57H	Choi *et al.*
	S171L	Truong *et al.*
	**T30I**	Choi *et al*., Borges *et al*., Truong *et al.*
E	N48D	Truong *et al.*
	S50I	Truong *et al.*
	**A2S/V**	Avanzato *et al*., Choi *et al.*
	R42K	Borges *et al.*
M	**H125S/Y**	Choi *et al*., Truong *et al.*
	S197T	Truong *et al.*
ORF6	V5I	Khatamzas *et al.*
	T39S	Borges *et al.*
	S83L	Choi *et al.*
ORF7a	Q94K	Choi *et al.*
	A105V	Truong *et al.*
ORF7b	Δ2	Bazykin *et al.*
	K2N	Avanzato *et al.*
ORF8	T11I	Truong *et al.*
	Q18STOP	Bazykin *et al.*
	L84S	Avanzato *et al.*
	T148A	Truong *et al.*
N	R195G	Bazykin *et al.*
	A208S	Choi *et al.*
	T325K	Khatamzas *et al.*
	N345K	Avanzato *et al.*

*Mutations in bold have been found to have arisen in multiple isolates.

### Deletions of the S1/S2 furin cleavage site emerge in cell culture as well as *in vivo* and attenuate the virus in airway cells and animal models of transmission

Vero E6 cells have been widely used for isolation and growth of SARS-CoV-2 stocks as they are readily available, easy to use and highly permissive to the virus [72]. However, during propagation of SARS-CoV-2 isolates in Vero cells, deletions incorporating, or flanking, the furin cleavage site between S1 and S2 spike subunits are often reported [43, 73–78]. Similar deletions have been detected in clinical samples at very low frequency, including from human autopsy samples [79–81]. The deletion of the furin cleavage site adapts the virus to higher replication in cells lacking TMPRSS2, such as Vero cells, but attenuates the virus in TMPRSS2-expressing cells such as primary human airway cells [51, 80, 82–85]. Furthermore, furin cleavage site deletions result in lower pathogenicity in animal models and attenuated virus transmission in hamster and ferret models [75, 80, 81, 84, 86]. This has implications for source virus used in infection and challenge studies and virus stocks should be sequenced prior to use to ensure the furin cleavage site is intact. We and others have suggested the furin cleavage site allows rapid TMPRSS2-dependent cell entry at the cell surface or early endosome allowing the virus to evade highly restrictive endosomal IFITM proteins (such as IFITM2 or IFITM3). In contrast, in the absence of TMPRSS2 the virus must enter via the endosome/lysosome to be activated by cathepsins. In the harsh conditions of the acidifying endosome or the lysosome, having a pre-cleaved S1/S2 site may be disadvantageous as it results in instability of the spike glycoprotein and premature S1 shedding [80, 85].

### The globally emerging strain first found in the UK, B.1.1.7, contains the spike glycoprotein mutation N501Y and shows signs of having emerged from an immunocompromised host

In December 2020, a cluster of COVID-19 cases (known variously as B.1.1.7, 20B/501Y.V1, or VOC/202012/01) was detected in South East England [87]. This cluster showed evidence of higher transmissibility in the community compared to contemporary strains [88], significantly higher case-fatality rates [89, 90], and, depending on the study, lower Ct values from diagnostic PCR tests on clinical swabs [91–94]. In the UK, B.1.1.7 is now the predominant lineage, accounting for >90 % of infections [95]. In many countries, initially imported B.1.1.7 is rapidly outcompeting local strains and becoming the major circulating strain [96–99]. B.1.1.7 contains seven non-synonymous mutations in the spike glycoprotein and a total of 23 mutations across the whole genome (see Table 2). Notable mutations in the spike glycoprotein include N501Y, Δ69–70, Δ144, and P681H in S1. N501Y lies in the RBD and has been described as increasing human ACE2 binding as well as enabling binding to mouse ACE2 [62, 100–102]. The P681H polymorphism lies adjacent to the S1/S2 furin cleavage site and we have shown this mutation alone, or in the B.1.1.7 spike, enhances its efficiency of furin cleavage [52]. The Δ69–70 and Δ144 deletions lie in the NTD region of spike and may modulate antigenicity [103–105]. Additionally, the B.1.1.7 lineage contains a premature stop codon in the accessory protein ORF8 and a three amino acid deletion in NSP6 (both described in more detail later in this review). The apparent long phylogenetic branch length and pattern of mutations has led to the hypothesis that this virus may have emerged from long-term infection in an immunocompromised patient, before spilling back into the general population [87]. There is growing evidence that B.1.1.7 viruses are only minimally less well neutralised by pre-B.1.1.7 convalescent or post-vaccine antisera [52, 66, 106–112]. Indeed vaccine effectiveness in the UK, where B.1.1.7 predominates, remains high [113].

**Table 2.** Substitutions and deletions seen in currently circulating variants of concern and variant of concern-like viruses

Variant names and aliases	Spike mutations	Other non-synonymous mutations in genome
B.1.1.7 VOC-20DEC-01 aka 20B/501Y.V1 (UK)	L18F*,†**, Δ69–70**, **Δ144, N501Y,** A570D, *D614G*‡, **P681H**, T716I, S982A, D1118H	NSP3 – T183I, A890D, I1412T; **NSP6 – Δ106–108**; NSP12 – *P323L*; ORF8 – Q27STOP, R52I, Y73C; N – D3L, S235F
B.1.351 aka 20B/501Y.V2 aka VOC-20DEC-02 (South Africa)	L18F*, D80A, D215G*, Δ242–244*, R246I*, K417N*, E484K, **N501Y,** *D614G*, **A701V**	NSP2 – T85I; NSP3 – K837N; NSP5 – K90R; NSP6 – Δ106–108*; NSP12 – *P323L*; ORF3a – *Q57H*, S171L; E – P71L; N – T205I
P.1 aka 20B/501Y.V3 aka VOC-21JAN-02 (Japan ex Brazil)	**L18F**, T20N§, P26S, D138Y, R190S*, **K417T, E484K**, **N501Y**, *D614G*, H655Y, T1027I	NSP3 - S370L, K977Q; **NSP6 – Δ106–108**; NSP12 – *P323L*; NSP13 – E341D; Orf3a – S253P*; ORF8 – E92K; N –P80R *R203K, G204R*
A.23.1/E484K aka VUI-21FEB-01 (UK)	R102I, F157L, V367F, **E484K**, Q613H, **P681R**	NSP3 – L741F; NSP6 – M86I, L98F, M183I; ORF8 – *L84S*, E92K; N – S202N
B.1.525 aka VUI-21FEB-03 (UK ex West Africa)	Q52R, A67V*, **Δ69–70, Δ144, E484K,** *D614G*, **Q677H**, F888L	NSP3 – T1189I; **NSP6 – Δ106–108;** NSP12 – P323F; E – L21F; M – I82T; ORF6 – Δ2; N – Δ2/D3Y, A12G, *T205I*
B.1.1.318 aka VUI-21FEB-04 (UK ex West Africa)	T95I, **Δ144**, **E484K,** *D614G,* **P681H, D796H**	**NSP1 – Δ85**; NSP3 – S126L*, E378V, K1693N; NSP4 – T173I, A446V; NSP5 – T21I; **NSP6 Δ106–108**; NSP12 – *P323L*; NSP15 – V320M; NSP16 – A116S*; M – I82T; ORF7b/ORF8 fusion +ORF8 E106STOP; N –*R203K, G204R*, Δ208/R209G
B.1.324.1/E484K aka VUI-21MAR-01 (UK ex Antigua)	**E484K,** S494P, **N501Y,** *D614G,* **P681H,** E1111K	NSP2 – T85I; NSP3 – T1378P*; NSP4 – T189I, T439M; NSP6 – H11Q; NSP9 – P57S; NSP12 – S6L, *P323L*; ORF3a – *Q57H*, ORF8 – 35 bp deletion and frameshift; N – M234I
P.3 aka VUI-21MAR-02 (Philippines)	**Δ141–143**, Δ243–244*, Y265C, **E484K, N501Y**, *D614G*, **P681H,** E1092K, H1101Y, V1176F	NSP3– D736G, S1807F*; NSP4 – D217N*, L438P; NSP6 – D112E; NSP7 – L71F; NSP12 – *P323L*;NSP13 – L280F*, A368V; ORF8 – K2Q; N – *R203K, G204R*
B.1.526 (New York)	L5F, T95I, D253G, E484K* or S477N*, *D614G,* **A701V,**	NSP2 – T85I; NSP3 – V1139I; NSP4 – L438P; NSP6 – L37F, **Δ106–108**, NSP12 *– P323L*; NSP13 – Q88H; ORF3a – P42L, *Q57H*; ORF8 – T11I; N – P199L, M234I
A.27 (Mayotte)	L18F*, L452R*, N501Y, A653V, **H655Y**, Q677H*, D796Y, G1219V	NSP2 – P106L; NSP4 – D217G*; NSP6 – N82S; NSP13 – P77L; ORF3a – V50A, 8nt deletion and frameshift; ORF8 – *L84S,* del119-120; *N – S202N*
Cluster 5 (Danish Mink)	**Δ69–70**, Y453F, *D614G*, I692V, M1229I	**NSP1 - Δ85;** NSP3 - Δ1264; NSP12 – *P323L*, T739I; NSP15 – T112I; ORF3a – H182Y; N - S194L, *R203K, G204R*

*Indicates mutation found in some, but not all variants of this lineage.

†Residues in bold indicate mutations found in multiple variants.

‡Residues in italics indicate substitutions likely present in the ancestral viruses.

§Indicates mutations predicted to result in addition of a new N-linked glycosylation site.

Persistent infection in immunocompromised patients may allow viruses to rapidly generate diversity under prolonged selection pressures that are absent in typical SARS-CoV-2 infections that transmit within days and resolve within weeks. Such infections have been proposed to be a potential mechanism for rapid antigenic evolution in influenza [114]. Various NTD deletions in the SARS-CoV-2 spike glycoprotein are commonly observed in immune-suppressed patients with a long-term infection, supporting the idea of intra-host evolution (see Table 1) [71, 100, 103, 104, 115–118]. A recurrent NTD deletion in the 140–145 region has been found in nine separate chronically infected patients, indicating this may be a signature mutation of these long-term infections [71, 100, 104, 115–118].This mutation is also found in the B.1.1.7 lineage (as well the similar B.1.318, B.1.525, and P.3 lineages, discussed later). These 140-loop deletions have additionally been suggested to allow escape from NTD-targeting antibodies and encompasses part of a large neutralising epitope known as the NTD antigenic ‘supersite’ [104, 105, 119].

Furthermore, the Δ69–70 deletion in the NTD has also arisen multiple times independently, both in healthy and immunocompromised humans, as well as in mink, and often in combination with RBD interface mutants as described earlier in this review [61, 71, 103, 104, 120]. It has been hypothesised that this deletion could act as a ‘permissive’ mutation, somehow allowing or compensating for receptor binding mutations (such as N439K, Y453F or N501Y) that alone may be deleterious to virus fitness, due to a currently undescribed effect on spike stability or similar [120]. A mechanism for this relationship between NTD and RBD is unclear at present as residues 69–70 of the NTD are distal to the RBD in both the open and closed spike trimer conformations [24].

The 69–70 deletion removes six nucleotides that are part of the probe target sequence in one of the commonly used RT-PCR tests used to screen swabs for diagnosis with COVID-19. The resulting ‘S gene target failure’ has been a fortuitous way to easily monitor the growth of lineages carrying this deletion, such as the UK B.1.1.7 variant of concern [91]. Fortunately, the diagnosis of cases has not been compromised because of the redundancy built into the diagnosis platforms that use several different primer-probe sets across the SARS-CoV-2 genome.

### Emerging variants first described in South Africa and Brazil contain the RBD mutation E484K which allows significant escape from human convalescent antisera

In recent months, several independent lineages of viruses containing the spike glycoprotein mutation E484K have been detected worldwide – once in South Africa (B.1.351 or 20B/501Y.V2) and at least twice independently in Brazil (P.1 or 20B/501Y.V3, and P.2) [121–123]. This mutation is of particular concern as independent studies have suggested E484K is a bona fide escape mutant to many convalescent antisera [108, 119, 124]. Both Brazil (particularly the Amazonas region) and South Africa experienced high disease burdens in 2020 and likely have high seroprevalence which may have driven emergence of these antigenic variants [125, 126]. This is further reinforced by several case studies showing E484K containing variants reinfecting healthcare workers in Brazil and a high rate of reinfection of seropositive individuals in the placebo arm of a vaccine trial in South Africa [122, 126–129]. Concerningly, recent evidence suggests that these E484K variants likely partially or fully escape vaccine- or naturally immunity-derived antisera [66, 108, 111, 130].

These E484K lineages represent three independent emergences of the same mutation: E484K alone in the P.2 lineage, or remarkably, together with K417T/N and N501Y in both the B.1.351 and P.1 lineages. Multiple independent lineages gaining the same or similar patterns of mutations and rapidly increasing in frequency strongly suggests positive selection and parallel, or convergent evolution. The E484K mutation combined with the N501Y mutation has been suggested to synergistically enhance binding of the spike glycoprotein to human ACE2 [62, 102]. Furthermore, the B.1.1.7 lineage, the P.1 lineage, and several isolates from the B.1.351 lineage all contain an identical deletion of amino acids 106–108 in NSP6, known as the ‘SGF deletion’ (Table 2). NSP6 is a multi-pass transmembrane protein that is thought to be involved in autophagy and antagonism of innate immune responses, but it remains unclear what influence this deletion has on virus phenotype [131]. Finally, several clusters of the B.1.1.7 variant in the UK contain E484K (currently known as VOC 202102/02) and this single mutation allows B.1.1.7 to escape from neutralising antisera raised after vaccination or infection with first wave strains of virus [112, 132].

### Repeated, independent emergence of SARS-CoV-2 variants of concern show signs of convergent evolution on a global scale

As well as the three main variants of concern described above (B.1.1.7, P.1, and B.1.351), in recent weeks further variants have been detected with similar amino acid substitutions throughout their genomes. The rapidly expanding list includes the B.1.526 lineage, first isolated in New York [133], the A.23.1(E484K) lineage found in the UK (and related to the A.23 and A.23.1 lineages found in Uganda) [53, 54], the B.1.525 and B.1.1.318 lineages, both seen in multiple countries but linked with travel to West Africa [134], the A.27 lineage, associated with a cluster of cases on the island of Mayotte [135], the B.1.341.1 lineage [136], seen in the UK and related to travel to Antigua, and finally the P.3 lineage described first in the Philippines [137, 138]. Although there is not currently strong evidence of more rapid transmission of most of these variants, they share a number of molecular characteristics with the major variants of concern, such as combinations of receptor avidity-enhancing mutations (N501Y, E484K and/or S477N), furin cleavage site adjacent mutations (P681R/H, Q677H, H655Y) and genomic deletions (spike ~Δ140 deletions, ~Δ243 deletions, and NSP6 Δ106–108, see Table 2). The similarity between many of these variants suggests a remarkable degree of convergent evolution.

### N439K and L452R are antigenic variants that spread rapidly in 2020

Since March 2020, the spike mutation N439K has arisen multiple times (all alongside D614G), independently in Europe and the USA. N439K lies directly within the RBD/ACE2 binding interface. Subsequent binding studies have shown this variant shows a modest increase in ACE2 binding and clinical data indicates marginally lower Ct values in clinical diagnostic PCR tests, indicative of higher replication [40]. Furthermore, N439K moderately alters antigenicity with some human convalescent antisera and monoclonal antibodies less able to bind and neutralise the variant virus or pseudovirus, although these <fourfold differences for polyclonal antisera are unlikely to have a major impact on vaccine effectiveness [35, 40]. The predominant lineage containing N439K (B.1.258) is now mostly found in combination with the NTD deletion Δ69–70, although the significance of this is currently unknown [120].

In July 2020 a pair of sister lineages of SARS-CoV-2 (B.1.427 and B.1.429) were first detected in California containing the RBD mutation L452R (as well as a pair of mutations in the NTD). Subsequently these lineages have become widespread across the USA [139]. Like N439K, L452R has been shown by several studies to reduce convalescent antisera binding [124, 140], however others have since shown this reduction only has a minimal effect on vaccine-induced neutralising antibody titres, particularly when compared to the E484K-containing variants of concern [66]. L452R is also present in several emerging variants, such as the A.27 lineage (Table 2).

### A222V and D839Y have emerged multiple times in the field and require further investigation

Although many spike glycoprotein variants have increased in frequency, phylogenetic analysis can show they are often the products of founder effects and it is therefore not clear whether they truly represent ongoing evolution and adaptation or are merely genetically neutral [33]. Two mutations outside of the RBD that have arisen multiple times and spread more widely than would be expected for neutral mutations are A222V, in the NTD, and D839Y, in the fusion peptide [39, 141]. There is speculation the main A222V-containing lineage (B.1.177) likely spread widely, particularly in the UK, due to a founder effect from tourists visiting Spain during the summer [141]. Unlike D614G, there was no evidence D839Y led to lower Ct values from swabs, suggesting this mutation doesn’t have a large impact of virus replication within the host [39].

## SARS-CoV-2 mutations outside the spike glycoprotein

Although the majority of focus on polymorphisms in SARS-CoV-2 has been on the spike glycoprotein and its importance as the major antigen of the virus, a number of other interesting polymorphisms have been described throughout the rest of the genome, some of which have been phenotypically characterised.

### Deletions in ORF8 have been associated with milder clinical disease

Like other coronaviruses, SARS-CoV-2 expresses several small accessory proteins. Whilst historically these proteins were not essential for cell culture propagation of coronaviruses, subsequently many of these proteins were found to have immune-modulating functions *in vivo* that may be host specific, therefore variation in such genes might be expected as SARS-CoV-2 adapts to optimal replication and transmission in humans [131].

ORF8 encodes an accessory protein of 121 amino acids that has been proposed to supress the immune response during infection, potentially by downregulating host MHC class I expression [142]. Several independently emerging SARS-CoV-2 variants containing deletions in ORF8 have been described. One large cluster from Singapore in the Spring of 2020 contained a 382-nucleotide deletion resulting in a truncated ORF7b and complete ablation of ORF8 expression [143, 144]. This strain was associated with milder infection and improved disease outcomes [145], but subsequently became extinct as Singapore implemented strict biosecurity measures. In human primary nasal epithelial cells the deletion of ORF8 resulted in a modestly delayed replication kinetics at early time points but little or no difference in transcriptional profile compared to wild-type virus [146].

Interestingly, the aforementioned UK B.1.1.7 lineage, assumed to have arisen from an immunocompromised individual, contains a premature stop codon at position 27 of ORF8 – it is highly likely this results in loss of function of the accessory protein, similar to the Singapore cluster [87, 145, 146]. However, unlike the Singapore cluster, B.1.1.7 has been found to have a higher case fatality rate than other circulating lineages [89, 90], potentially due to the complex set of mutations in this variant of concern. Additionally, a single case report from an immunocompromised patient also included a premature stop codon in ORF8, similar to the B.1.1.7 lineage [71].

At the equivalent genome position of SARS-CoV-2 ORF8, SARS-CoV contains open reading frames for a pair of small accessory proteins, ORF8a and ORF8b. SARS-CoV animal isolates, as well as early human isolates, contained a single open reading frame (ORF8) at this position but later human isolates encoded ORF8a and ORF8b after a 29nt out-of-frame deletion in the gene [147, 148]. It is unclear if there is a link between this apparent SARS-CoV human adaptation and the recurrent SARS-CoV-2 ORF8 deletions seen, as the ORF8 proteins of the two viruses are highly divergent but this serves as an example of how accessory proteins might evolve during human adaptation.

### Deletions and truncations of ORF7a have arisen multiple times independently

ORF7a is another accessory protein of SARS-CoV-2 and is thought, like its SARS-CoV orthologue, to interfere with surface expression of host restriction factor tetherin and to modulate host translation [149, 150], although others have recently suggested this tetherin inhibition may be a non-specific side effect of ORF7a-mediated Golgi fragmentation [151]. Several independent studies have detected SARS-CoV-2 isolates with unique in- and out-of-frame deletions in this protein resulting in heavily truncated versions being expressed [152–156]. It has been hypothesised that due to possible redundancy between ORF7a and ORF6 of SARS-CoV-2, which also is thought to also inhibit host translation, deletions in ORF7a may come at a low fitness cost *in vivo* [154]. Presently, no work has been published investigating what impact, if any, these deletions have on virus fitness.

The B.1.318 variant described previously, through a combination of nonsense mutations and a deletion of the intergenic region, is predicted to express a 146 amino acid ORF7b/ORF8 fusion protein – composed of the entire ORF7b protein fused to amino acids 3–105 of ORF8. It is unclear at present what the function or significance of this fusion protein might be, however it is by mechanisms such as this that we might expect novel accessory proteins to arise in coronaviruses.

### Viruses with truncated ORF6 proteins result in a more pro-inflammatory innate immune response

ORF6 is another accessory protein involved in blocking the innate immune response. ORF6 of both SARS-CoV and SARS-CoV-2 can block nuclear import of STAT [157]. Multiple SARS-CoV-2 isolates have been found with truncations in ORF6, including in a nosocomial cluster, suggesting the viruses are able to transmit, at least in a hospital setting [158–160]. Virus replication kinetics of the variants with ORF6 truncations are equivalent to that of closely related isolates with intact ORF6 (160). However, the truncation does appear to result in an increase in NF-κB related innate inflammatory responses [159].

### The P323L mutation of the viral polymerase; an important human adaptation or a genetic hitchhiker?

The major catalytic component of the SARS-CoV-2 RNA-dependent RNA polymerase (RdRp) is the NSP12 subunit. NSP12 is a typical viral RdRp containing an serine-aspartic acid-aspartic acid (SDD) catalytic site and is the catalytic subunit in the replication/transcription complex responsible for viral RNA synthesis. The P323L polymorphism co-arose in the same virus cluster as the now dominant D614G mutation in the spike glycoprotein (lineage B.1 and its progeny lineages), along with a pair of non-coding/synonymous changes in the 5′ UTR and NSP3 (30). Position 323 lies distal to the NSP12 catalytic core and is therefore unlikely to directly influence polymerase enzymatic activity. Rather this residue is located at the NSP12 surface, proximal to one of the binding sites for NSP8, a small polymerase cofactor. It is possible this mutation could be modulating NSP8 interaction or interaction with a yet unknown viral or host factor. Although reverse genetics experiments have shown D614G alone provides greater entry into human cells and greater viral fitness in animal models [41, 44, 45], it remains unclear if this mutation alone entirely recapitulates the phenotype seen with the whole virus isolates that also contain P323L. Further experiments are needed to determine whether P323L may also be playing a role in the success of this virus genotype or whether it is simply a genetic hitchhiker propelled by the success of the spike glycoprotein D614G substitution.

The B.1.525 lineage, which has many variants of concern-like properties and has been associated with travel to West Africa, contains the further NSP12 mutation, L323F. The significance of a further onward mutation at this position is currently unclear; a direct proline to phenylalanine substitution at this position would require a minimum of two nucleotide changes which is unusual, even in RNA viruses. Therefore, it is possible this mutation signifies fine tuning of this position to further enhance viral fitness, or that this is a partial reversion of a detrimental hitchhiker mutation. Either way emphasises position 323 plays an important, but undefined, role in polymerase function. *In vitro* assays with constituted polymerase could be of use to decipher this question, as might studies with SARS-CoV-2 replicons [131, 161].

### Other rarer deletions of attenuating or unknown phenotype

As well as the mutations described above, several less common but notable deletions have been observed in SARS-CoV-2 sequences, most of which are currently phenotypically uncharacterised.

From analysis of 17 928 genome sequences, a nine-nucleotide deletion (amino acids Δ241–243) was identified in NSP1 and was found in multiple different geographical locations. The role of NSP1 is to dampen the immune responses through host shut-off by inhibiting host ribosomal translation [162]. This deletion is hypothesised to destabilise the C-terminal domain, potentially impacting viral replication and leading to a less pathogenic phenotype [163]. Furthermore, a single amino acid deletion in NSP1 (Δ85) has been described in several variants of concern-like viruses or immunocompromised patient sequences (Tables 1 and 2) [61, 115].

Viral genomes harbouring deletions can be found alongside wild-type genomes within a single patient. A 12-nucleotide deletion has been identified in the SARS-CoV-2 E in a clinical isolate; however, the mutant was rare in clinical samples and emerged *in vitro* during cell culture passage. E is a small transmembrane, structural protein involved viral envelope formation and maturation. Both the mutant and wild-type were able to infect Vero cells and produced comparable viral titres, but the mutant virus isolate had higher spike glycoprotein content [164]. As the mutant was not identified in clinical samples, it is possible the deletion event is a result of cell culture adaptation. However, there are other reports of clusters of clinical isolates from India containing comparable deletions in E [165]. It has been shown previously that SARS-CoV artificially engineered to lack E gene expression is highly attenuated *in vitro* and *in vivo* [166].

Analysis of recombination at the minor variant level in a cohort of patients from the UK, show evidence of deletion events throughout the viral genome, particularly in ORF3a and ORF7a; accessory proteins that are both involved in interferon responses. These deletions may act as defective genomes [167]. Such deletion variants may contribute to the transient, within host emergence of SARS-CoV-2 isolates associated with milder disease [145].

### The potential roles of host-mediated RNA-editing in SARS-CoV-2 evolution

Mutations in virus genomes are usually considered to be a result of error-prone viral polymerases; however, host factors can also play a role in the editing of the viral genome. The two main host candidates for endogenous mutagenesis are the RNA-editing enzymes of the APOBEC and ADAR families [168]. APOBEC proteins are interferon-stimulated and responsible for the deamination of cytosine to uracil after replication and before packaging of virus particles [169–171], whereas ADAR proteins are responsible for the deamination of adenine to inosine driving A ->G changes during replication [172]. For influenza virus, we have previously shown that apparent RNA editing can result in the rapid emergence of antigenic variants with multiple concurrent amino acid changes [173]. Studies have shown a bias in C ->U mutations within the SARS-CoV-2 genome, suggesting editing by APOBEC [168, 171, 174–179]. RNA editing is enriched in putative RNA loop regions, presumably due to being more exposed than other parts of the genome [177]. Due to observations of a bias in C ->U mutations in the SARS-CoV-2 genome, it has been suggested that cytidine rich regions should be avoided during the design of diagnostic tests [178].

Furthermore, it has also been shown that virus-derived RNA sequences enriched for Uracil correlate with enhanced production of pro-inflammatory cytokines when comparing to the sequence of a reference virus. Based upon previous studies showing U-rich ssRNA stimulating the innate immune response through TLR7 signalling [180, 181], Kosuge *et al.* (2020), investigated the impact of C ->U point mutations on the host response, showing an increase in TNF-α and IL-6 production in immune cell lines [176].

## Conclusions

Although SARS-CoV-2 has only circulated in humans for a little over a year, an unprecedented sequencing effort has led to the description of many variants. Until recently, the only robust evidence for a genotypic change that had a strong phenotype was the spike mutation D614G that has strongly been selected for in the human population. D614G has been shown to enhance virus entry and replication in the human respiratory tract. It is now necessary to understand which adaptive mutations are enhancing transmission and driving the increase of new variant of concern lineages such as B.1.1.7, now predominant in the UK and spreading worldwide, and whether the E484K mutation observed in multiple independent lineages from areas across the world that have encountered multiple waves of high virus circulation, including South Africa and Brazil, as well as a subset of the B.1.1.7 lineage, may allow antigenic escape and jeopardise vaccine efforts [119, 121, 123, 124, 127, 132]. The recent rise of new virus lineages underscores the importance of continued sequencing efforts to rapidly identify new variants. At present, a subset of countries, such as Denmark and the UK, are contributing a disproportionate amount of the total SARS-CoV-2 genome sequences, thanks largely to pre-existing sequencing capacity and recent public funding. Consequently, these countries have often been the first to describe these new, imported variants, however in many areas it is very unclear what strains and lineages are circulating. Ideally viruses must be sequenced from as broad a possible range of countries and regions and the results made available in a timely manner for surveillance of new variants to have significant policy impacts.

Several studies have examined potential antigenic variants using either ‘reverse genetics’ approaches - mostly generating libraries of pseudotypes with naturally occurring changes or changes in predicted antigenic regions - or ‘forward genetics’ approaches – using authentic virus, replication competent chimeric viruses (generally vesicular stomatitis virus with its native glycoprotein replaced with SARS-CoV-2 spike), or phage/yeast display screens and selecting with antibodies to drive the emergence of variants in either naturally occurring or mutagenesis-derived quasi-species or mutant libraries [35, 119, 124, 182, 183]. These approaches are a key part of our ability to predict the antigenic effect of mutations. However, although a variety of mutants have been shown to escape neutralisation or binding from monoclonal antibodies, and occasionally convalescent antisera, it is still unclear whether these mutations would come with a fitness costs in the context of infectious viruses that would make them less likely to arise in the field. Furthermore, although the majority of approved vaccines specifically target the humoral immune response against the spike protein, further work is needed to understand the potential role of cellular immunity in natural infection and how this could be used to optimise future vaccines.

As the level of natural immunity increases and global mass vaccination intensifies, it becomes ever more important to continuously sample, sequence and antigenically characterise novel virus variants, particularly from reinfections or from those who have been vaccinated [126]. This will allow for rapid detection of antigenic variants that could lead to potential vaccine failure, and for rapid vaccine updates where required, in a similar manner to seasonal influenza. The possibility of antigenic drift is something vaccine designers, regulators and manufacturers should prepare for in the coming months and years. A likely scenario based on the development of animal coronavirus vaccines is that future SARS-CoV-2 vaccines may have to be multivalent to protect against multiple circulating antigenic variants, similar to vaccines against influenza or the avian gammacoronavirus infectious bronchitis virus in poultry [184].

While changes in the spike protein are most important antigenically, genome alterations that change expression of viral accessory proteins are also expected and may influence transmission and pathogenicity. During its relatively brief human circulation, SARS-CoV ORF8 quickly gained a deletion leading to the creation of ORF8a and ORF8b [147]. Multiple SARS-CoV-2 isolates with accessory protein deletions and truncations are already described, these alterations can also occasionally lead to the expression of ‘fusion ORFs’ encompassing the N-terminus of one protein and the C-terminus of another. These variants remain rare and none has yet spread rapidly in the human population; most have quickly died out with the exception of the truncated ORF8 seen in the globally emerging B.1.1.7 lineage. However, this clearly remains an area that should be closely monitored during surveillance and performing whole genome sequencing (rather than just spike sequencing) remains imperative, since this type of genetic change might have an impact on transmission or clinical outcomes.

In addition, surveillance for reverse zoonoses and for mutations in chronically ill people with COVID-19 should be reinforced and intensified. Certain companion and farmed animals are clearly highly susceptible to SARS-CoV-2 and their infections could drive the selection of variant viruses with different receptor binding or antigenic properties that could cross back into humans. Similarly, the rapidly spreading B.1.1.7 lineage in the UK, which is hypothesised to have gained multiple mutations in a chronically ill patient, is only one of several variants reported from such individuals displaying multiple markers of potential altered antigenic and receptor binding properties, as well as a higher infectivity than previously circulating strains [100, 103]. Constant monitoring and enhanced biosecurity measures in these groups are essential to avoid novel virus variants from emerging.

It is important to note that, whilst consensus genomes are reported in global databases, individuals are infected with and have a population of virus within them [167], manifesting as a consensus genome and minor variants, and all of these genomes are subject to selection pressure. Individual variations can be selected if they are advantageous or through founder effect or a mixture of both. In viruses with significant deletions in proteins, or the presence of stop codons, reported at a consensus sequence level, these may be balanced within the virus population with the presence of functional proteins at a minor variant level, similar to that observed in Ebola virus infection in humans [185]. Deep sequencing of clinical isolates can reveal important cooperative interactions between members of the virus population within a single host, and a better understanding of transmission bottlenecks will allow us to understand whether such interactions are perpetuated in transmission chains.

To summarise, there is an urgent need to continue to perform in-depth surveillance and sequencing of SARS-CoV-2 isolates over the coming months and years coupled with detailed downstream phenotypic analysis of the impact of mutations in near real time. This analysis should include both traditional techniques with mutant virus isolates and closely related controls which can be performed fairly rapidly, as well as more modern, but slower, techniques such as reverse genetics [186], which are vital to disentangle the phenotypes of mutations that occur across multiple genes.
